# Continuous cell supply from Krt7-expressing hematopoietic stem cells during native hematopoiesis revealed by targeted *in vivo* gene transfer method

**DOI:** 10.1038/srep40684

**Published:** 2017-01-18

**Authors:** Yoko Tajima, Keiichi Ito, Ayumi Umino, Adam C. Wilkinson, Hiromitsu Nakauchi, Satoshi Yamazaki

**Affiliations:** 1 Division of Stem Cell Therapy, Center for Stem Cell and Regenerative Medicine, The Institute of Medical Science, The University of Tokyo, Minato-ku, Tokyo, Japan; 2 Institute for Stem Cell Biology and Regenerative Medicine, Stanford University School of Medicine, 265 Campus Drive, Stanford, California 94305, USA

## Abstract

The nature of hematopoietic stem cells under normal hematopoiesis remained largely unknown due to the limited assays available to monitor their behavior *in situ*. Here, we develop a new mouse model to transfer genes specifically into the primitive hematopoietic stem cell compartment through the utilization of a modified Rcas/TVA system. We succeeded in transferring a GFP reporter gene into adult hematopoietic stem cells *in vivo*, which are predominantly quiescent, by generating pseudotyped-lentivirus. Furthermore, we demonstrate the utility of this system to study neonatal hematopoiesis, a developmental stage that has been difficult to analyze to date. Using the system developed in this study, we observed continuous multi-lineage hematopoietic cell supply in peripheral blood from Krt7-positive hematopoietic stem cells during unperturbed homeostatic condition. This powerful experimental system could provide a new standard tool to analyze hematopoiesis under physiological condition without transplantation.

Hematopoietic stem cells (HSCs) are one of the most well-studied adult stem cell populations and have provided many important concepts in the stem cell biology field^1^,^2^,^3^,^4^,^5^. HSCs are a rare cell population found within the bone marrow (BM), which possess remarkable functional capacity: multi-lineage differentiation potential and life-long self-renewal capability. This has led to the general view that HSCs reside at the apex of the hematopoietic cell hierarchy and are the life-long source of all adult hematopoietic cells. These functional characteristics of HSCs have been largely validated by the use of transplantation assays^6^. However, this assay exposes cells to various stresses that can be considered as non-physiological^7^,^8^. Therefore, the functions and dynamics of HSCs under steady state and non-transplantation conditions have remained largely obscured.

In contrast to HSCs, many other adult stem cell populations have been validated through *in vivo* genetic lineage tracing approaches, rather than transplantation assays^9^,^10^. The most successful cases include the identification of the intestinal stem cells using genetic lineage tracing of Lgr5-expression with the Cre/LoxP recombination system^11^. Recently, a similar lineage tracing approach has been undertaken to study steady state HSCs using Tie2 driven MerCreMer mice^12^. These experiments suggested fundamental differences between the function of HSCs in the maintenance of the hematopoietic system and in its re-establishment after transplantation.

An alternative method used to genetically label specific cell populations *in situ* is the Rcas/TVA system, in which the avian leucosis retrovirus (Rcas) is used to specifically infect cells that ectopically express the tumor virus A (TVA) antigen^13^,^14^,^15^. This system provides a powerful tool to analyze gene function and cell fate *in vivo*. The generation of mice expressing the *tva* gene under the control of a tissue- or cell type-specific promoter and their *in vivo* transduction by injection of Rcas virus into mice have been reported^16^,^17^,^18^,^19^. However, the use of this system has mostly been limited to actively proliferating cell types, such as those from neonatal stages or cancer models, since cell proliferation is required for the efficient infection of the Rcas retrovirus. Although the introduction of an oncogene into adult mammary epithelial cells *in vivo* by injecting lentivirus directly into mammary ductal lumen has been reported^20^, the application of the Rcas/TVA system to study normal adult stem cell populations has not been successful to date.

Here, we report the establishment of an HSC-specific *in vivo* gene transfer method, based on a modified Rcas/TVA system, for the study and perturbation of steady state adult hematopoiesis. We overcome two major obstacles, namely the generation of HSC-specific TVA-expressing mice and the generation of high-titer lentivirus that is capable of infecting TVA-expressing cells *in vivo* regardless of their cell cycle status. We focused on *Krt7* as a potential marker for HSCs, and by using the system developed in this study, we confirm long-term multi-lineage hematopoiesis from a Krt7-expressing adult cell population *in vivo*, suggesting that Krt7-positive cells exist within the HSC population and serve as a source of mature blood cells in native, unstressed conditions.

## Results

### Krt7 is expressed in HSC fractions within the hematopoietic system

**Figure 1:**
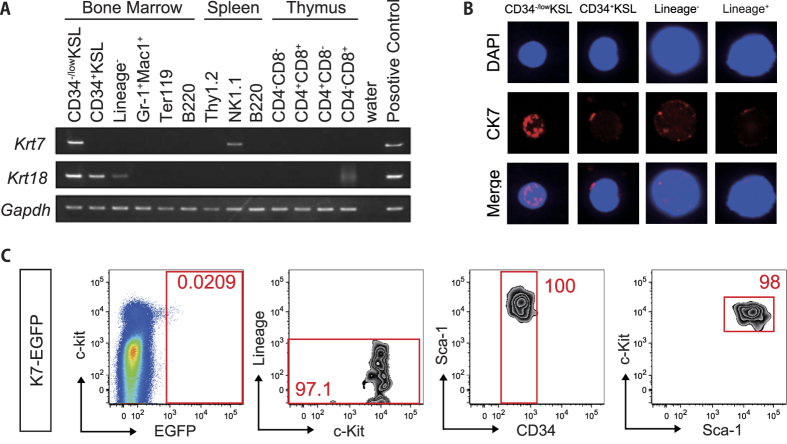
Krt7 expression in hematopoietic lineage. **(A)** RT-PCR analysis of *Krt7, Krt18* and *Gadph* (control) gene expression from various FACS-purified hematopoietic cell populations. Data representative of three independent experiments. CD34^−/low^KSL represents hematopoietic stem cell (HSC) fraction, CD34^+^ KSL represents progenitor fraction and Lineage^−^ represents undifferentiated fraction in bone marrow. **(B)** Representative immunohistochemical staining of single CD34^−/low^KSL (n = 70), CD34^+^ KSL (n = 7), Lineage^−^ cell (n = 9) and Lineage^+^ (differentiated) cell (n = 9). Sorted cells were stained with Cytokeratin 7 (CK7, protein expressed from *Krt7* gene) antibody (*red*). Nuclei were stained with DAPI (*blue*). **(C)** Flow cytometeric BM analysis of chimera mice derived from *Krt7-EGFP* knock-in (K7-GFP) ES cells. Data representative of three individual mice.

Previous research by our laboratory identified Cytokeratin 18 (*Krt18*, type I member of the keratin superfamily) phosphorylation to be specific to self-renewing HSCs. To investigate whether any cytokeratin family members displayed HSC-specific gene expression, we undertook RT-PCR in different hematopoietic cell fractions. While *Krt18* was broadly expressed in hematopoietic progenitors, *Krt7* expression was specific to the HSC fraction (CD34^−/low^KSL) (Fig. 1A, Fig. S1A,B). *Krt7* was also more highly expressed in fetal liver (CD150^+^ KSL) HSCs than other fetal liver hematopoietic populations (Figure S1C). Krt7 is a presumptive type II assembly partner for Krt18 that has not previously been described to play a role in HSCs. By in-droplet staining, we further confirmed protein level expression of Krt7 in the majority of HSCs (75.8 ± 0.58%), which was not seen in later HPC populations (Fig. 1B). In order to visualize the expression pattern of *Krt7* at the cellular level *in vivo*, we generated a *Krt7-EGFP* knock-in embryonic stem (ES) cell line and analyzed GFP expression within the BM of chimeric mice generated by blastocyst injection of *Krt7-EGFP* ES cells (Fig. S1C–E). The GFP^+^ cells were highly enriched within the CD34^−/low^KSL population, confirming that *Krt7*-expression highly correlated with phenotypic HSC identity (Fig. 1C). While *Krt7* mRNA expression was detectable in NK1.1^+^ spleen cells, we could not detect Krt7-EGFP protein level expression within this population. These data suggest Krt7 can be used as an HSC-specific marker.

### Generation of HSC-specific TVA expressing mice

**Figure 2:**
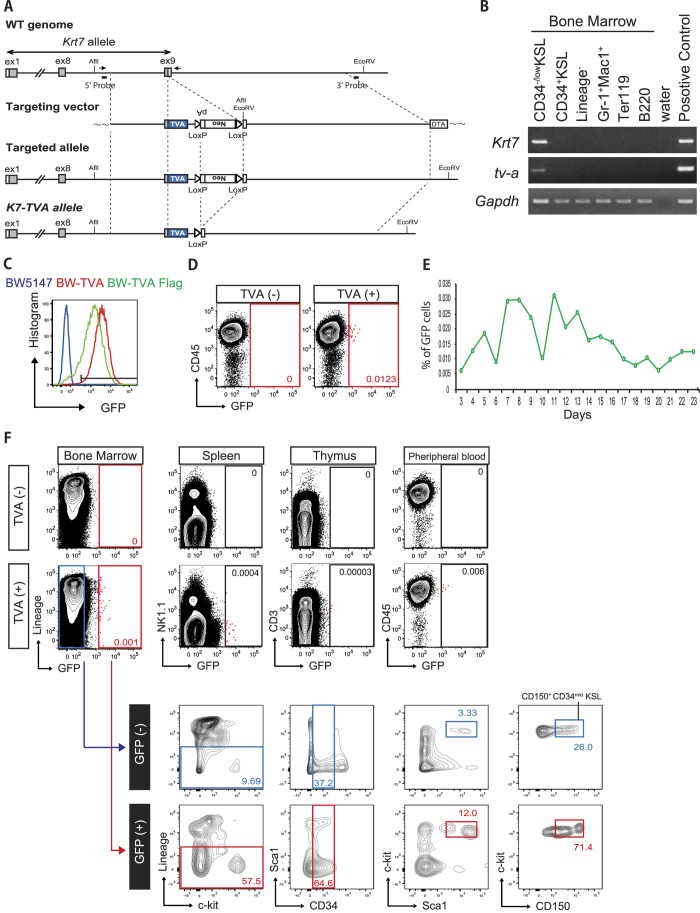
Generation of Krt7-TVA mice and *in vivo* gene transfer. **(A)** Targeting strategy for the *Krt7-TVA* knock-in (K7-TVA) mice. The upstream and downstream fragments (total 10 kb) of the stop codon of *Krt7* were subcloned into targeting vector as the 5′- and 3′-arm, respectively. T2A peptide sequence followed by TVA construct was designed to insert at 3′ end of Krt7 transcript. Restriction enzymes and Probes (shown as the *thick bar*) for Southern blotting are depicted. Arrows represent the primers designed for genotyping. **(B)** RT-PCR analysis of *tva, Krt7* and *Gapdh* gene expression in FACS-purified hematopoietic cell populations from K7-TVA mice. **(C)** GFP expression TVA transgenic mouse lymphoma cells (BW-TVA in red, BW-TVA Flag in green) and parental line (BW5147 in blue) three days after Rcas/GFP retrovirus transduction. **(D–F)** *In vivo* gene transfer by Rcas/GFP virus. Long-term analysis of intraperitoneal Rcas/GFP virus injected neonatal K7-TVA mice and TVA(−) littermate controls. **(D)** Representative flow cytometric plots displaying analysis of peripheral blood at 22 weeks post-injection. GFP^+^ cells only detected in TVA(+) littermates. **(E)** Percentage of GFP^+^ cells in CD45^+^ PB population, collected weekly over a six months period. The data shows one representative individual out of five TVA(+) K7-TVA mice. **(F)** Flow cytometric plots displaying analysis of GFP^+^ cells in various hematopoietic organs at 24 weeks post-injection. CD150^+^ CD34^−/low^KSL represent primitive hematopoietic stem cell fraction (considered a more highly purified stem cell fraction than CD34^−/low^KSL). Data representative of five mice.

Having identified *Krt7* expression to highly correlate with phenotypic HSC, we next leveraged this knowledge to establish an HSC-specific *in vivo* gene delivery method, based on the Rcas/TVA system. The Rcas retrovirus specifically infects cells expressing the TVA antigen through its viral envelope protein envA. We first aimed to generate HSC-specific TVA-expressing mice by targeting the avian *tva* gene into the *Krt7* locus in ES cells (Fig. 2A, Fig. S2A).

During the course of this research, Kataoka *et al*.^21^ demonstrated that Evi1 expression marks HSCs (although is also expressed in the hematopoietic progenitor cell fraction)^21^. Therefore, we also targeted the *tva* gene into the *Evi1* locus, to serve as a positive control for these experiments (Fig. S2DE). From these ES cell gene targeting experiments, we generated two HSC-TVA knock-in mouse lines by ES cell blastocyst injection: Krt7-TVA (termed K7-TVA) and Evi1-TVA (Fig. S2A,E). These mice were born normally, appeared healthy and displayed normal fertility. Flow cytometric analysis did not identify any obvious changes in appearance of the stem cell fraction, the BM hematopoietic lineages, or the peripheral blood (PB) between TVA^+^ and TVA^−^ mice (Fig. S2B,C,G,H). We further confirmed that *tva* mRNA expression correlated with that of the targeted gene locus (*Krt7* and *Evi1*) in the TVA knock-in mice (Fig. 2B, Fig. S2F).

### Production of Rcas/GFP retrovirus and *in vivo* gene transfer

We next prepared Rcas virus harboring a constitutive GFP reporter (Rcas/GFP) and confirmed that the Rcas/GFP virus infected only avian cells but not mammalian cells in culture (Fig. S3A). To check the dependence of Rcas/GFP transduction on TVA expression, we generated transgenic BW5147 mouse lymphoma cell lines expressing TVA: BW-TVA and BW-TVA-Flag. TVA expression resulted in high Rcas/GFP transduction efficiency of BW5147 cells (Fig. 2C).

To determine the infection ability of Rcas/GFP virus *in vivo*, we intraperitoneally injected the virus into neonatal K7-TVA mice, in which HSCs are still proliferative. Three weeks after the infection, we genotyped the mice and analyzed GFP expression in the peripheral blood (PB) by flow cytometry. As predicted, we observed GFP^+^ cells in PB of TVA^+^ mice but not TVA^−^ mice injected the virus (Fig. 2D). Weekly flow cytometric analysis revealed that GFP^+^ cells could be detected for over 6 months following Rcas virus injection in multi-lineage of hematopoietic cells (Gr1^+^ Mac1^+^ monocytes, B220^+^ B cells, CD3^+^ T cells) (Fig. 2E, Fig. S3B). These results implied stable integration of the GFP reporter gene into neonatal hematopoietic stem/progenitor cells that had the ability to form multiple hematopoietic cell lineages in adult mice. Next, we analyzed GFP expression within hematopoietic organs (BM, spleen, and thymus) of K7-TVA mice, to trace the potential supply origin of the GFP^+^ cells identified in the PB (Fig. 2E). We identified GFP^+^ hematopoietic cells at very low frequency in all hematopoietic organs. Notably, GFP^+^ cells also existed within the primitive HSC fraction of the BM (Fig. 2F). These results suggest that the Rcas retrovirus in combination with the K7-TVA mice provides a novel system to genetically label and/or perturb neonatal hematopoietic stem/progenitor cells.

### Generation of a high titer Rcas-based lentivirus for *in vivo* gene transfer

We next attempted to transduce adult HSCs *in vivo* by using tail vein injection of Rcas/GFP retrovirus into 8–12 week-old adult K7- or Evi1-TVA mice. However, GFP^+^ cells were not detected in BM or PB in either HSC-TVA line, even after one-year post-injection. While the Rcas virus displayed high titer when tested using BW-TVA cells, this retroviral required cellular proliferation for transduction. Since *tva* expression was detectable (Fig. 2B, Fig. S2F), these results suggested that TVA expression in the adult was confined to quiescent cells (a characteristic associated with the primitive HSC compartment *in vivo*) that were resistant to retroviral transduction.

To overcome this technical limitation, a cell-cycle independent viral transduction system was needed. We therefore generated and tested a modified envA pseudotyped GFP-expressing lentivirus, which utilizes the envA-TVA specificity of the Rcas virus transduction system, but affords cell-cycle independent lentiviral transduction^22^. While envA/GFP lentivirus also displayed dependence on TVA for transduction of BW5147 cells, only low titer was possible, which was not suitable for *in vivo* gene transfer (Table 1; ASLV-envA, ASLV-VCT). This limited virus generation could be due to the low *envA* expression from the pCMMP plasmid or envA protein translation and/or folding efficiency.

**Figure 3:**
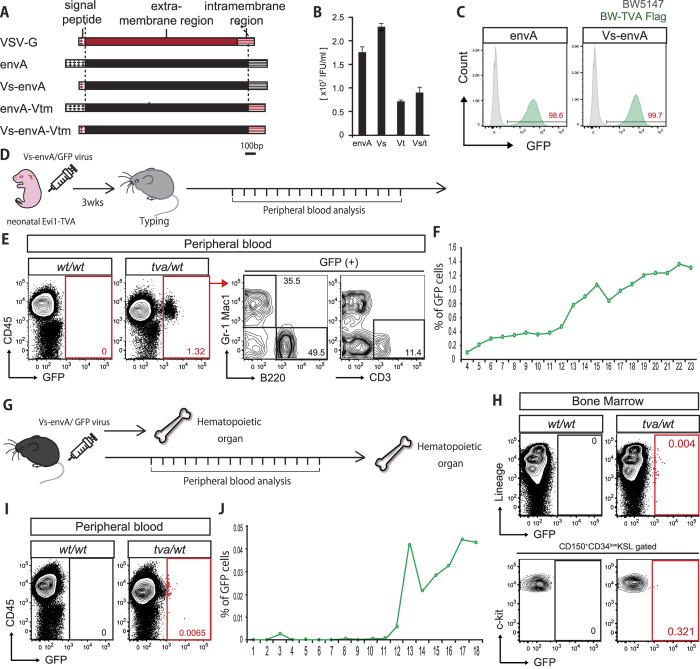
Generation of Vs-envA lentivirus and *in vivo* gene transfer. (**A**) Design of modified envA lentivirus envelope proteins using signal sequence and/or intramembrane region of the VSV-G envelope protein. (**B**) Lentiviral titer generated using the modified enviA envelope proteins described in A. Results are the mean ± S.D. from three independent experiments. **(C)** GFP expression of BW5147 and BW-TVA cells three days after transduction with modified envA lentiviruses. **(D)** Experimental procedure of *in vivo* gene transfer in neonatal Evi1-TVA mice. Vs-envA lentivirus was injected into the intraperitoneal cavity of neonatal (postnatal day 0–3) Evi1-TVA(+) mice and TVA(−) littermates, and GFP expression was followed in the PB from 3 weeks onwards. **(E)** Representative flow cytometric plots displaying analysis of peripheral blood in Evi1-TVA mice (injected Vs-envA/GFP virus at neonatal stage) at 21 weeks post-injection. GFP^+^ cells detected only in TVA(+) littermates, and within multiple lineages (Gr1^+^ Mac1^+^ monocyte, B220^+^ B cells and CD3^+^ T cells). **(F)** Percentage of GFP^+^ cells in CD45^+^ PB population Evi1-TVA mice (injected with Vs-envA/GFP virus at neonatal stage), collected weekly over a six months period. The data shows one representative individual out of four TVA(+) Evi1-TVA mice. **(G)** Experimental procedure of *in vivo* gene transfer in adult Evi1-TVA mice. Vs-envA lentivirus was injected via the tail vein of 8–12 week-old Evi1-TVA(+) mice and TVA(−) littermate controls. **(H)** Flow cytometric plots displaying GFP expression within the bone marrow at 7 days post-injection. **(I)** Representative flow cytometric plots displaying analysis of PB from Evi1-TVA mice (injected Vs-envA/GFP virus at adult stage) at 15 weeks post-injection. GFP^+^ cells detected only in TVA (+) littermates. **(J)** Percentage of GFP^+^ cells in CD45^+^ PB population Evi1-TVA mice (injected Vs-envA/GFP virus at adult stage) over a four month period. Data for one representative individual out of four TVA(+) Evi1-TVA mice.

In order to improve the titer of this lentiviral system, we next tried expressing *envA* from a pCMV plasmid, and also designed three modified versions of envA (Fig. 3A). The G protein of the Vesicular Stomatitis Virus (VSV-G) envelope protein affords very high lentivirus titer generation. We hypothesized that replacing the signal peptide and/or intramembrane region of the envA with that of VSV-G may improve lentivirus generation, while maintaining the extramembrane domain that is responsible for the TVA-dependent infection. We compared the transfection efficiencies and specificities of the four lentiviruses (envA and modified envA lentiviruses) using BW-TVA Flag and BW5147 cells. While transduction efficiency varied (Fig. 3B and Table 1; RCAS-envA ~ RCAS-Vs-envA-Vtm), all lentiviruses show transduction specificity for TVA expression (Fig. 3C). The Vs-envA protein, in which the envA signal sequence replaced by the VSV-G counterpart, showed the highest titration and was therefore taken forward for *in vivo* analysis.

To initially assess the infection capabilities of Vs-envA/GFP lentivirus *in vivo*, we intraperitoneally injected the virus into neonatal Evi1-TVA mice (Fig. 3D). We analyzed PB of injected mice by flow cytometry every week from four weeks after birth to over six months of age. GFP^+^ cells were detected from the first analysis (four weeks) and continuously observed over the subsequent five months (Fig. 3E,F). GFP^+^ cells were present in multiple hematopoietic lineages (Fig. 3E), indicating that neonatal hematopoietic stem/progenitor cells were labeled by the Vs-envA/GFP lentivirus and their progeny were continuously contributing mature blood cells to the PB long-term.

### *In vivo* gene transfer in adult hematopoietic stem/progenitor cells

Having confirmed the *in vivo* transduction capacity of the Vs-envA/GFP lentivirus to genetically label neonatal hematopoietic stem/progenitor cells, we next applied this lentivirus system to investigate native adult HSCs. As a positive control, we initially injected the Vs-envA/GFP lentivirus into the tail vein of adult Evi1-TVA mice (Fig. 3G). After the first week of injection, we analyzed the BM by flow cytometry (Fig. 3H). While only a very small percentage of the BM expressed GFP, a few GFP^+^ cells were detected within the primitive stem cell fraction (CD150^+^ CD34^−^KSL). GFP expression was also tracked in the PB weekly for four months post-injection (Fig. 3I,J). GFP^+^ cells were detected in PB (Fig. 3I), where they appeared from around 12 weeks post-injection (Fig. 3J). GFP^+^ cells were detected continuously from this time point, suggesting that the Vs-envA lentivirus could successfully transfer reporter genes into adult hematopoietic stem/progenitor cells and that their progeny could stably inherit the GFP reporter.

**Figure 4:**
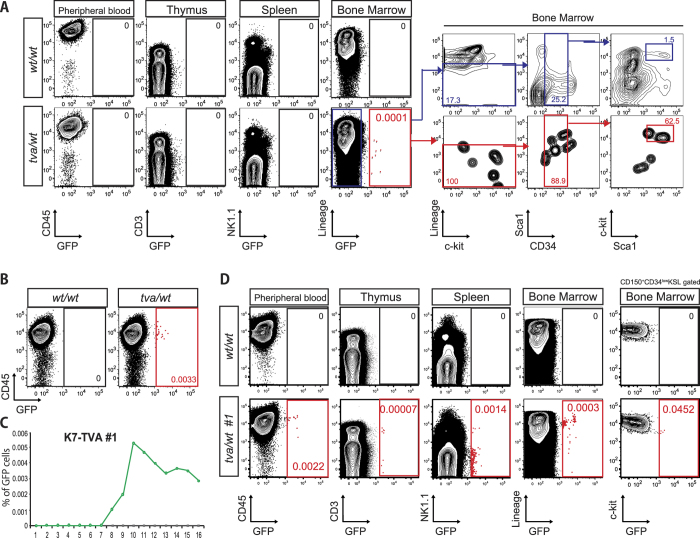
Hematopoietic stem cell specific gene transfer *in vivo*. **(A–D)** *In vivo* gene transfer in adult K7-TVA mice. Vs-envA lentivirus was injected via the tail vein of 8–12 week-old K7-TVA(+) mice and TVA(−) littermate controls. **(A)** Flow cytometric plots displaying analysis of GFP expression in the hematopoietic organs (peripheral blood, thymus, spleen and bone marrow) of K7-TVA mice 7 days post-injection. Note GFP^+^ cells exist only in bone marrow of TVA(+) mice. Flow cytometric gating for the hematopoietic stem cell fraction in the bone marrow shown on the right. Upper right panel displays the pattern of GFP^−^ (untransduced) cells. The lower right panel displays the pattern for GFP^+^ (transduced) cells, which shows high enrichment in the CD34^−/low^KSL fraction. **(B)** Representative flow cytometic plots displaying GFP expression in the PB of K7-TVA mice (injected Vs-envA/GFP virus at adult stage) at 15 weeks post injection. GFP^+^ cells detected only in TVA(+) mice. **(C)** Percentage of GFP^+^ cells in CD45^+^ PB population K7-TVA mice (injected Vs-envA/GFP virus at adult stage) over a four-month period. **(D)** Flow cytometric plots of GFP expression as described in A, but at 17 weeks post-injection. All data representative of at least four mice.

Finally, we assessed the *in vivo* gene transfer using the Vs-envA lentivirus and K7-TVA mice. We injected Vs-envA/GFP virus into the tail vein of adult K7-TVA mice. One-week post-injection, we analyzed the hematopoietic organs for GFP expression by flow cytometry. Interestingly at this time point, GFP^+^ cells were only detected in the BM but not in PB, spleen and thymus (Fig. 4A). Although the percentage of GFP^+^ cells in the BM was extremely low (0.0001–0.01%), GFP^+^ cells mainly existed in the CD34^−/low^KSL fraction. We also analyzed PB of K7-TVA mice injected with Vs-envA/GFP virus every week over a four-month period. GFP^+^ cells became detectable in the PB at around 8 weeks post-injection from which time they were continuously detectable (Fig. 4B,C and Fig. S4D. Importantly, GFP^+^ cells were detected in multiple hematopoietic lineages (Gr1^+^ Mac1^+^ monocytes, B220^+^ B cells and CD3^+^ T cells) within the PB. To help trace the origin of these GFP^+^ cells, we sacrificed these mice 17 weeks post-injection and analyzed the hematopoietic organs by flow cytometry (Fig. 4D). At this time point, GFP^+^ cells were observed in all hematopoietic organs analyzed (thymus, spleen and BM), although still at low percentages. Moreover, GFP^+^ CD150^+^ CD34^−^KSL cells were detectable, a likely long-term source of GFP^+^ cells in the PB (Fig. 4D). These data suggest the possible path by GFP^+^ HSCs in BM to mature blood cells in the PB.

## Discussion

To our knowledge, this is the first demonstration of stable gene transfer into HSCs *in vivo*. We achieved this by combining newly generated HSC-TVA mice lines and modified envA lentivirus (Fig. S4E). Importantly, this system that does not use transplantation, irradiation or chemical treatment, allowing the use study of native HSCs *in vivo*. While we have validated this novel assay using a GFP reporter gene to conducted lineage-tracing experiments *in vivo*, essentially any lentivirus construct can be used, giving this system broad applications in the study of HSCs.

Following HSC emergence from E10.5 within the mouse embryo, HSCs migrate to and rapidly proliferate within the fetal liver before seeding the BM around the time of birth, where they progressively mature into a largely quiescent population of adult stem cells^23^. While adult HSCs have been a focus of much research, fetal and neonatal HSCs have remained more elusive, in part due to their migratory patterns. During the validation of the HSC-TVA mice, we unexpectedly captured neonatal HSCs *in vivo*. The HSC-specific expression of Krt7 (both fetal and adult stages) means that this system therefore has potential to identify, track and perturb HSCs as they develop and mature within the embryo and neonatal mouse. Although we also detected weak *Krt7* expression within NK1.1^+^ cells, we did not identify GFP expression within NK1.1^+^ cells following virus injection (Fig. 4A), suggesting that the *Krt7* promoter did not drive high enough *TVA* expression in NK1.1^+^ cells for their *in vivo* transduction.

While proliferative neonatal HSCs and progenitors were efficiently transduced by Rcas retrovirus, quiescent adult HSCs were resistant to transduction. Through adoption and optimization of a lentivirus envA system, we have been able to transduce adult HSCs *in vivo*, without loss of TVA-dependent infection specificity. While the modified Rcas/TVA system has significant potential to track adult HSC dynamics *in vivo*, this system also has application for HSC perturbation within the context of native hematopoiesis, including its use in gain- and loss-of-function screening. Although our average infection ratio of adult HSCs was low, further improvement should be possible through larger scale virus production. However, this low transduction efficiency of adult HSCs may provide a significant advantage for disease modeling, such as leukemia, by providing a physiologically relevant model to investigate the progression of disease from rare oncogenic events. Mutational complexities such as order of mutation acquisition, which are now thought to be effect in disease progression^24^, could also be directly investigated through sequential *in vivo* transduction.

Recently, lineage-tracing experiments have been undertaken within the context of unperturbed hematopoiesis, (Busch *et al*.^12^, Sun *et al*.^25^). Both reports suggested that native HSCs provide only a minor contribution to steady-steady hematopoiesis, differing from the currently prevalent view of the hematopoietic hierarchy. Consistent with these recent reports, our results indicate that like transplanted HSCs, native HSCs also have multi-lineage potential, but the generation of mature hematopoietic cells is slower in the unperturbed setting: GFP^+^ cells were only detected in the PB after 2–3 months following envA/GFP injection into Evi1-TVA and Krt7-TVA mice. In comparison to these alternative lineage-tracing approaches, our approach has the advantage of affording native HSC perturbations. Screening using our *in vivo* transduction may well provide different results to those undertaken using HSC transplantation assays, and important biological insights.

We and others have recently validated other HSC-specific genes, including Fgd5 and Hoxb5^26^,^27^. However, Krt7 may be advantageous over these HSCs markers. Hoxb5 appears to be extremely specific to primitive HSCs but its expression is very low and required a triple mCherry tag for detectable expression, meaning *Hoxb5* may not be suitable to drive TVA expression. Fgd5 appears to have higher expression within HSCs but is also expressed in non-hematopoietic BM cell types (such as endothelial cells). Its use for driving TVA expression may therefore result in perturbation of the HSC niche, and indirectly alter HSC activity. However, it is important to mention that Krt7 and Evi1 are expressed in other, non-hematopoietic tissues. For example, we observed GFP^+^ cells in the pancreas, testis and intestine of Evi1-TVA mice following injection of Rcas/GFP virus. These TVA mouse lines may therefore have useful application to the study, transduction and perturbation of these other tissues.

In conclusion, we report the first demonstration of *in vivo* gene transfer into the most primitive hematopoietic cell compartment, and validate with novel insights into native hematopoiesis. These data highlight the broad potential of this approach for the investigation of hematopoietic system dynamics in development, homeostasis and disease.

## Methods

### Mice

C57BL/6 (B6-Ly5.2) and ICR mice were purchased from SLC (Shizuoka, Japan). Animal care in our laboratory was in accord with the guidelines of the University of Tokyo for animal and recombinant DNA experiments. We were conducted at The University of Tokyo, under an approved Institutional Animal Care and Use Committee protocol (no. A16-46). All animal experiments were carried out in accordance with the approved guidelines.

### Cell culture

BW5147 and 293 T cell lines were cultured in Dulbecco’s Modified Eagle Medium (Thermo Fisher Scientific) supplemented with 10% Fetal Bovine Serum (FBS) and 1% penicillin, streptomycin, glutamine (PSG), at 37 °C with 10% CO_2_. The DF-1 cell line was cultured as above but at 39 °C and with 5% CO_2_. ES cells (K3: 129SvxC57BL/6, mB1.2: C57BL/6) were cultured in Glasgow Minimum Essential Medium (Sigma) supplemented with 15% FBS, 1% non-essential amino acid (Invitrogen), 100 mM Sodium Pyruvate (Invitrogen), 110 μM 2-mercaptoethanol (Invitrogen), 1% PSG, 500 U/ml ESGRO LIF (CHEMICON), 3 μM CHIR99021 (Wako) and 1 μM PD0325901 (Wako) at 37 °C with 5% CO_2_ on gelatin-coated dishes seeded with mitotically-inactivated mouse embryonic fibroblasts.

### Molecular cloning

The *tva* gene was cloned from pCMMP-TVA800 vector (Addgene, Plasmid #15778). PCR-mutagenesis was used to generate TVA-Flag construct to insert a Flag tag downstream of the signal sequence of *tva*. The *envA* gene was cloned from RCAS/GFP vector (kindly provided by Dr. Fukuhara, Kumamoto University). Complete *envA* transcript were amplified by PCR and cloned into pCMV expression vector. At the same time, *vsv-g* derived from vesicular stomatitis virus and chimeric envelope of *envA* and *vsv-g* were cloned. We designed three chimeric envelope sequences that were generated by PCR. Vs-envA: Fusion of signal peptide of VSV-G and envA excluded its counterpart. envA-Vtm: Fusion of VSV-G intramembrane domain and envA excluded its counterpart. Vs-envA-Vtm: Fusion of signal peptide and intramembrane domain of VSV-G and envA excluded its counterpart.

### Generation of TVA expressing mouse lymphoma cell line

pGCDNsam retrovirus harboring TVA or TVA-Flag construct were generated using 293gp cells and used to infect BW5147 cells, from which clonal populations were derived. BW-TVA and BW-TVA-Flag expressing clones were identified by flow cytometry using a mouse anti-TVA IgG antibody that we generated in house. We would like to note that while the TVA antibody could detect TVA overexpression on BW-TVA and BW-TVA-Flag cell lines, it was not possible to detect TVA expression on primary cells.

### Virus production

The Rcas/GFP vector was transfected into DF-1 cells by calcium phosphate. Seven days after transfection, almost all the DF-1 cells expressed GFP. Rcas/GFP viruses were harvested from the culture media 10 days to 4 weeks post-transfection. Virus was concentrated by ultracentrifugation at 40,000 *g* for 3 hours, with the pellet being resuspended in 1/250 volume of a-MEM. Concentrated viruses were stocked at −80 °C and titer measured using BW-TVA or BW-TVA-Flag cells. envA and modified envA lentivirus were produced using 293 T cells. Envelope expression vectors were cotransfected with pMDL-gag/pol and CS-CDF-CG-PRE vectors using polyethylene imine^28^. The culture media was refreshed after 12–16 hours with media supplemented with 10 uM Forskolin. After 48 hours, virus was collected, stored and tested as above.

### Isolation of hematopoietic lineages

We sampled bone marrow, spleen and thymus of 8–12 week old mice. We made a single cell suspension and stained with the following antibody cocktails and analyzed by flow cytometry. Bone marrow: Gr-1 PE, Mac1 Pacific Blue, Ter119 FITC (APC), B220 PE-Cy7. Spleen: Thy1.2 PE, NK1.1 FITC (APC), B220 PE-Cy7. Thymus: CD3 FITC (APC-Cy7), CD4 PE, CD8 APC. For hematopoietic stem cell analysis, CD150 PE, CD34 FITC (Pacific Blue), c-kit APC, Sca-1 PE (PE-Cy7) and biotin-tagged lineage marker antibodies (Gr-1, Ter119, CD4, CD8, B220 and IL-7 receptor) were used in combination with Streptavidin APC-Cy7. In this report, CD34^−/low^ c-kit^+^ Sca-1^+^ lineage^−^ (CD34^-/low^KSL) or CD150^+^ CD34^−/low^KSL fractions were treated as the hematopoietic stem cell fraction.

### RT-PCR and qPCR

RNA was extracted using the RNeasy Micro Kit (QIAGEN Germantown, MD) from which cDNA was synthesized using High Capacity cDNA Reverse Transcription Kit (Thermo Fishier Scientific). The following positive controls were used in these experiments: mouse stomach cDNA (*Krt7* and *Krt18*); whole embryo cDNA (*Evi1*); TVA vector DNA (*tva*), and bone marrow c-Kit- cell cDNA (*Gapdh*). TaqMan reagent (PE Applied Biosystems, Foster City, CA) was used for quantification of Gapdh, and Universal Probe Library (Roche) for each gene of interest. Primer: *Krt7* Fwd: gatcaagaccctcaacaaca/Rvs: ccttcttcagcaacacaaac, *Krt18* Fwd: aagaggaagtccaaggtctg/Rvs: tcatggagtccaagtcaatc, *Gapdh* Fwd: cttcaccaccatggagaaggc/Rvs: ggcatggactgtggtcatgag. Q-PCR *Krt7* Fwd: tctttgaggctcagattgctg/Rvs: cgtcggttgatctcctcttc *Krt18* Fwd: gaagaaccgcgaggaactg/Rvs: cttggtggtgacaactgtgg.

### In droplet immunostaining

Glass slides with 4 mm diameter holes (Matsunami TF2404) were coated with 10% poly-L-lysine, washed with distilled water and dried. 20 μl of S-clone (Sanko-Junyaku, Tokyo, Japan) was applied each hole and hematopoietic cells were sorted onto the slide by fluorescence activated cell sorting. After 30–60 minutes, cells were fixed with 4% paraformaldehyde for 10 minutes at room temperature. Slides were then washed twice with PBS, and Max Block Blocking (Active Motif) applied for 30 minutes at room temperature. Cells were incubated with diluted anti-Cytokeratin 7 antibody (Abcam, ab9021), overnight at 4 °C. The next day, the cells were washed three times with PBS and incubated with diluted goat anti-mouse IgG H&L (Alexa Fluor 647), for 60 minutes at room temperature. After washing seven times with PBS, cells were stained with dihydrochloride (1 μg/ml) for 1 minute and washed again. Slides were then mounted with Fluorescent Mounting Medium (Dako), and imaged by confocal microscopy (Olympus).

### Generation of knock-in mice

pBl-lx Neo-DTA were utilized as the targeting vector. For Krt7-EGFP and Krt18-tdTomato knock-in vectors, a flexible linker of 5 Gly (GAA) codons followed by the fluorescence reporter gene was cloned into the stop codon of the cytokeratin gene, for expression as fusion protein. For the K7-TVA knock-in vector, a T2A peptide sequence followed by the tva-flag gene sequence were inserted into the stop codon of *Krt7* gene. The Evi1-TVA knock-in vector design was based on a Evi1-EGFP knock-in construct (Kataoka *et al*.^21^). We designed to insert Evi1 cDNA (exon10–15) fallowed by T2A peptide sequence and tva-flag transcript to end of exon9 of *Evi-1* gene. Each vector was constructed with total 10 kb arm of upstream and downstream of the targeted locus. 20 μg targeting vector were mixed with 1 × 10^6^ ES cells and electropolated. After two days of electroporation, G418 (300 μg/ml) were added in the culture. ES cell colonies with good morphology were picked, expanded and genotyped.

### Genotyping

Genomic DNA was extracted from ES cells or the tip of mouse-tail. For screening of ES cells, samples were resuspended in 750 μl Lysis buffer (1 M Tris-HCl/5 M NaCl/0.5 M EDTA/10% SDS/H_2_O) and incubated with Proteinase K 15 μl for 30 minutes at 55 °C, before incubation with 10 μl RNaseA (10 mg/ml) for 30 minutes at 37 °C. Samples were then mixed with 750 μl phenol/chloroform and centrifuged for 30 minutes at room temperature. The supernatant were again mixed with the same volume and centrifuged. Genomic DNA were purified from its supernatant by ethanol precipitation. PCR was undertaken using HS Ex-Taq (Takara), using 30 cycles of 98 °C for 10 seconds and 68 °C for 60 seconds.

The tip of mouse-tail were boiled with 180 μl of 50 mM NaCl for 15 minutes at 95 °C. The samples were neutralized with 20 μl of 1 M Tris-HCL (pH 7.0) and centrifuged 12,000 × *g* for 10 minutes. We used its supernatant as PCR template. PCR was undertaken as above but with 35 cycles of 98 °C for 10 seconds and 68 °C for 24 seconds. The following primers were used. K7-EGFP Fwd: gaagcctgctgattcctgac/Rvs: aagtcgtgctgcttcatgtg. K18-tdTomato Fwd: atcaacggaacaaaggatcg; Rvs: ggggaacttcctgactaggg. ES cell K7-TVA Fwd: ggcataggcatgggagtaga; Rvs: caccagctcacagcaaaaga. ES cell Evi-1-TVA Fwd: ctcgtcctgcagttcattca; Rvs: attcagggattttgctgcac. Tail K7-TVA Fwd: gttccctgggtaggggagt; Rvs: cgctctgttccctgtgagta; tcaccgcatgttagcagact. Tail Evi1-TVA Fwd: aaggggaaagagcgctacac; Rvs: cgatgaaattgcggatctct; Rvs: gttcaaatcccagcaaccac.

### *In vivo* gene transfer

For infection experiments against neonates, we peritoneal injected concentrated virus solution day 1~3 days after birth. Three weeks after injection, mice were genotyped and blood samples were taken every week for analysis. For adult, virus solution was injected into tail vein of 8–12 week old mice. Hematopoietic organs (bone marrow, thymus, spleen and peripheral blood) were analyzed at 7 days of post injection, or blood samples were taken every week for analysis. We used 200 μl (~0.8 × 10^7^ IFU) of concentrated Vs-envA/GFP virus solution (from 7 dishes of 293 T), and 200 μl (~0.8 × 10^7^ IFU) of concentrated Rcas/GFP virus solution for each injection.

## Additional Information

**How to cite this article**: Tajima, Y. *et al*. Continuous cell supply from Krt7-expressing hematopoietic stem cells during native hematopoiesis revealed by targeted *in vivo* gene transfer method. *Sci. Rep.* **7**, 40684; doi: 10.1038/srep40684 (2017).

**Publisher's note:** Springer Nature remains neutral with regard to jurisdictional claims in published maps and institutional affiliations.

## Supplementary Material

Supplementary Information

## Figures and Tables

**Table 1:** Comparison of Virus Titration.

Virus	Virus Type	Titration (IFU/ml)
Rcas/GFP	Retro	∼4.5 × 10^7^
ASLV-envA/GFP	Lenti	∼0.3 × 10^7^
ASLV-VCT/GFP	Lenti	∼0.2 × 10^7^
Rcas-envA/GFP	Lenti	1.8 × 10^7^ ± 1 × 10^6^
Rcas-Vs-envA/GFP	Lenti	2.3 × 10^7^ ± 8 × 10^5^
Rcas-envA-Vtm/GFP	Lenti	0.7 × 10^7^ ± 4 × 10^5^
Rcas-Vs-envA-Vtm/GFP	Lenti	0.9 × 10^7^ ± 1 × 10^6^
