# Supplementary material for: Continuous cell supply from Krt7-expressing hematopoietic stem cells during native hematopoiesis revealed by targeted in vivo gene transfer method
Source: Sci Rep. 2017 Jan 18;7:40684. doi: 10.1038/srep40684 (PMC5241640; doi:10.1038/srep40684)
Supplement: Supplementary Information [file srep40684-s1.pdf]

Supplementary Information

Continuous cell supply from Krt7-expressing hematopoietic stem cells during native hematopoiesis revealed by targeted in vivo gene transfer method

Yoko Tajima, Keiichi Ito, Ayumi Umino, Adam C Wilkinson, Hiromitsu Nakauchi and Satoshi Yamazaki

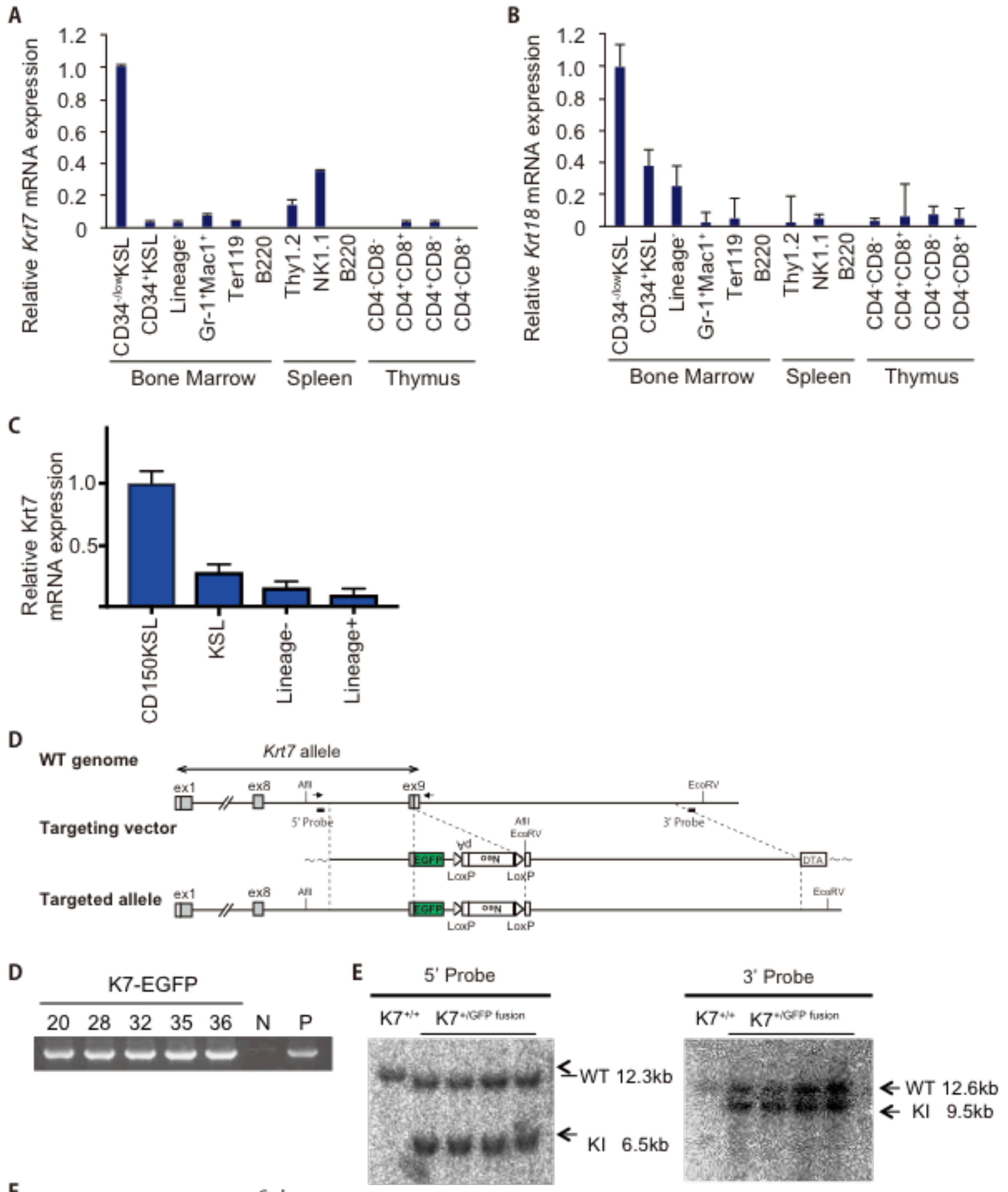

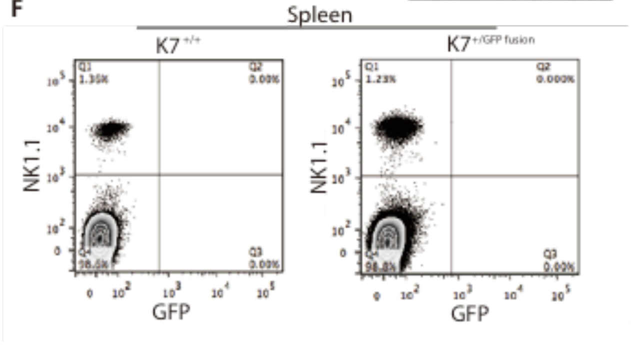

Fig. S1 Generation of K7-EGFP knock-in ES cells.

Figure S1, related to Figure 1: Generation of K7-EGFP knock-in mice

(A,B) Quantitative RT-PCR analysis of *Krt7* and *Krt18* gene expression (relative to *Gadph*) from various FACS-purified adult hematopoietic cell populations. The expression in CD34$^{-/low}$KSL (HSC fraction) was set at 1.0. Results are presented as the mean ± S.D. of triplicate samples from three independent experiments. **(C)** As in (A) but from fetal liver populations from E14.5 mouse embryos. **(D)** Targeting design for *Krt7-EGFP* knock-in mice. **(E)** PCR screening of *Krt7-EGFP* knock-in ES cells. **(F)** Southern blot of Krt7-EGFP knock-in ES cells. (**G**) Representative flow cytometric plots for NK1.1 and GFP in the spleen from WT and *Krt7-EGFP* mice (of three mice).

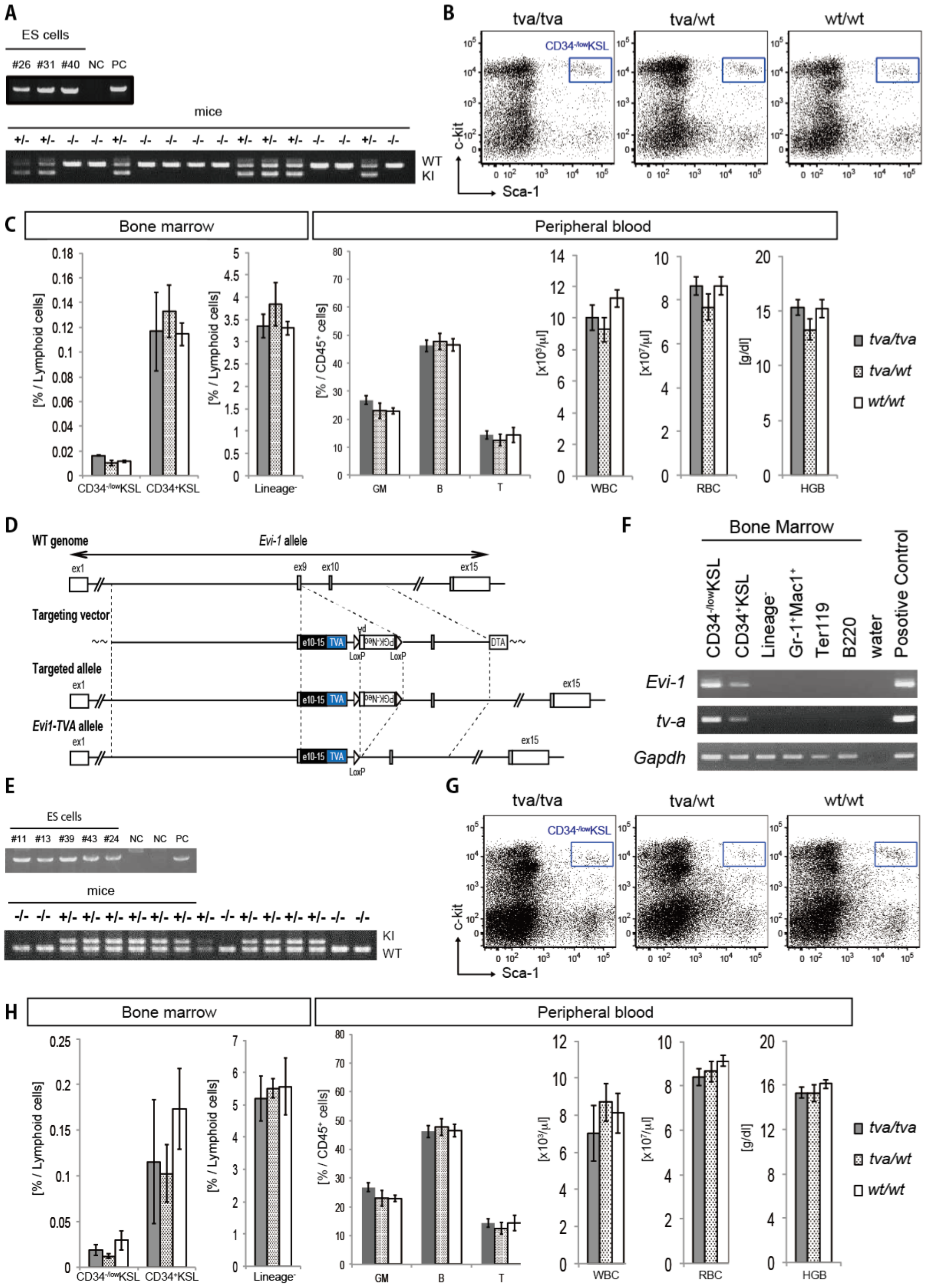

A
ES cells
#26 #31 #40 NC PC
mice
WT
KI
B
tva/tva
tva/wt
wt/wt
CD34-/lowKSL
c-kit
Sca-1
C
Bone marrow
Peripheral blood
[% / Lymphoid cells]
[% / CD45+ cells]
[x10³/µl]
[x10⁷/µl]
[g/dl]
CD34-/lowKSL
CD34+KSL
Lineage-
GM
B
T
WBC
RBC
HGB
tva/tva
tva/wt
wt/wt
D
WT genome
Evi-1 allele
Targeting vector
Targeted allele
Evi1-TVA allele
ex1
ex9
ex10
ex15
e10-15
TVA
PGK-Neo
pA
LoxP
DTA
E
ES cells
#11 #13 #39 #43 #24 NC NC PC
mice
KI
WT
F
Bone Marrow
CD34-/lowKSL
CD34+KSL
Lineage-
Gr-1+Mac1+
Ter119
B220
water
Posotive Control
Evi-1
tv-a
Gapdh
G
tva/tva
tva/wt
wt/wt
CD34-/lowKSL
c-kit
Sca-1
H
Bone marrow
Peripheral blood
[% / Lymphoid cells]
[% / CD45+ cells]
[x10³/µl]
[x10⁷/µl]
[g/dl]
CD34-/lowKSL
CD34+KSL
Lineage-
GM
B
T
WBC
RBC
HGB
tva/tva
tva/wt
wt/wt

Figure S2, related to Figure 1: Characterization of HSC-TVA mice

(A) PCR screening of K7-TVA knock-in ES cells and genotyping of K7-TVA knock-in mice. **(B)** Representative flow cytometric plots displaying gating for HSC in K7-TVA mice. Indicated fraction was gated with PI^{-} Lineage $marker^{-}$ $CD34^{-/low}$. The blue enclosing line HSC fraction ($CD34^{-/low}$c-kit^{+}Sca-1^{+}Lineage $marker^{-}$). Representative of five mice. **(C)** Comparison of hematopoietic lineage of K7-TVA knock-in mice. Percentage of $CD34^{-/low}$KSL, $CD34^{+}$KSL and $Lineage^{-}$ fraction per lymphoid cells in bone marrow, and Gr-1^{+} $Mac1^{+}$ monocyte, $B220^{+}$ B cells, $CD3^{+}$ T cells and blood count in peripheral blood. Data represent mean ± S.D. from three mice. **(D)** Targeting design for Evi1-TVA knock-in mice. **(E)** PCR screening of Evi1-TVA knock-in ES cells and genotyping of Evi1-TVA knock-in mice. **(F)** RT-PCR expression analysis of *tva* transcript on hematopoietic lineage in bone marrow of Evi1-TVA mice. **(G)** As in B, but for Evi1-TVA mice. Representative of three mice. **(H)** As in C, but for Evi1-TVA mice. Data represent mean ± S.D. from five mice.

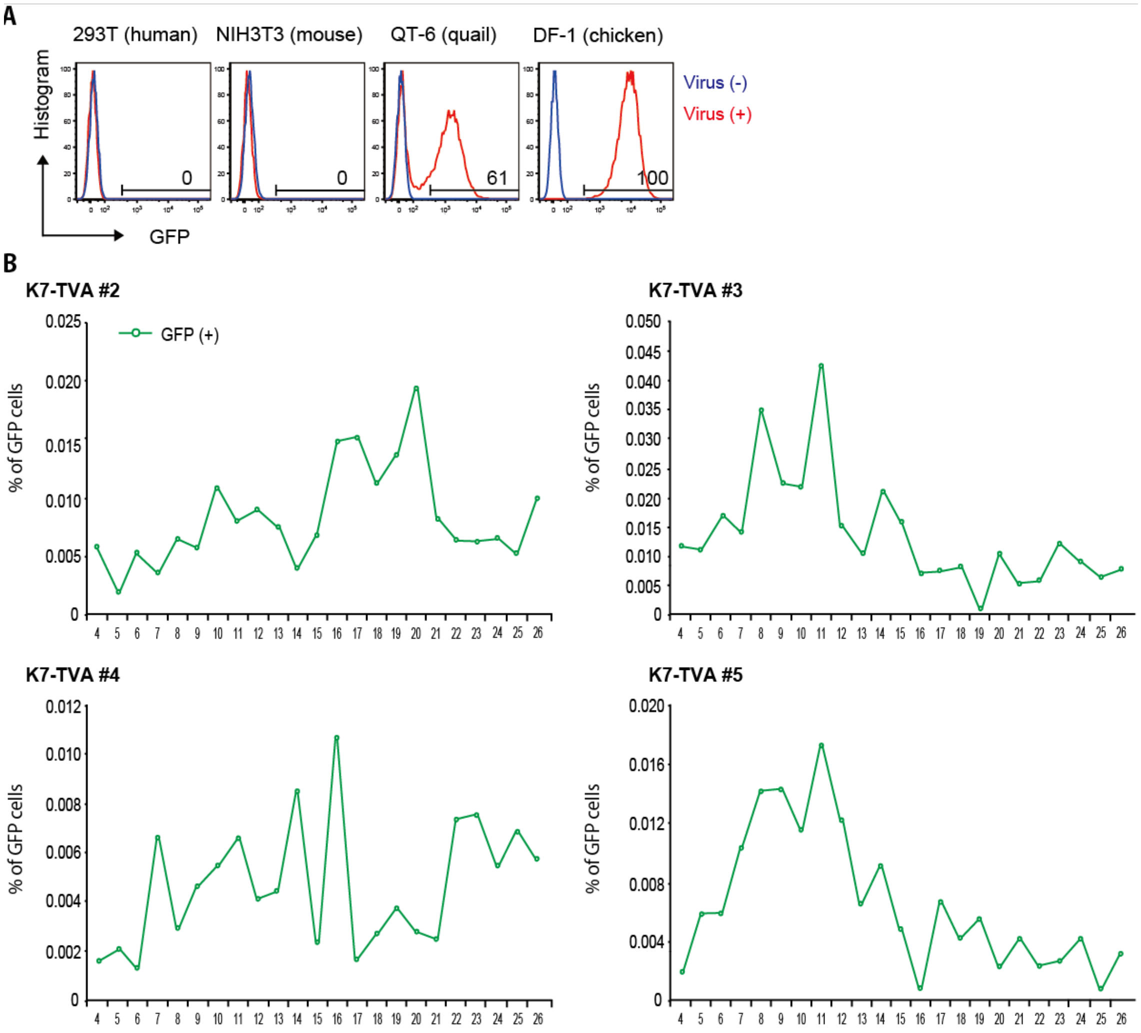

Figure S3, related to Figure 2: In vivo gene transfer at neonatal stage of K7-TVA mice

(A) Infection specificity of Rcas virus to avian cells. Rcas virus harboring GFP (Rcas/GFP) was added into the culture media of 293T (human), NIH3T3 (mouse), QT-6 (quail) and DF-1 (chicken) cell lines. Infection ability of the virus was determined by flow cytometric analysis of GFP expression 3 days after transduction. Data representative of three experiments. **(B)** *In vivo* gene transfer in neonatal K7-TVA mice (other individuals to that displayed in Fig.2E). Percentage of GFP^+ cells in the PB of K7-TVA mice injected Rcas/GFP virus at neonatal stage.

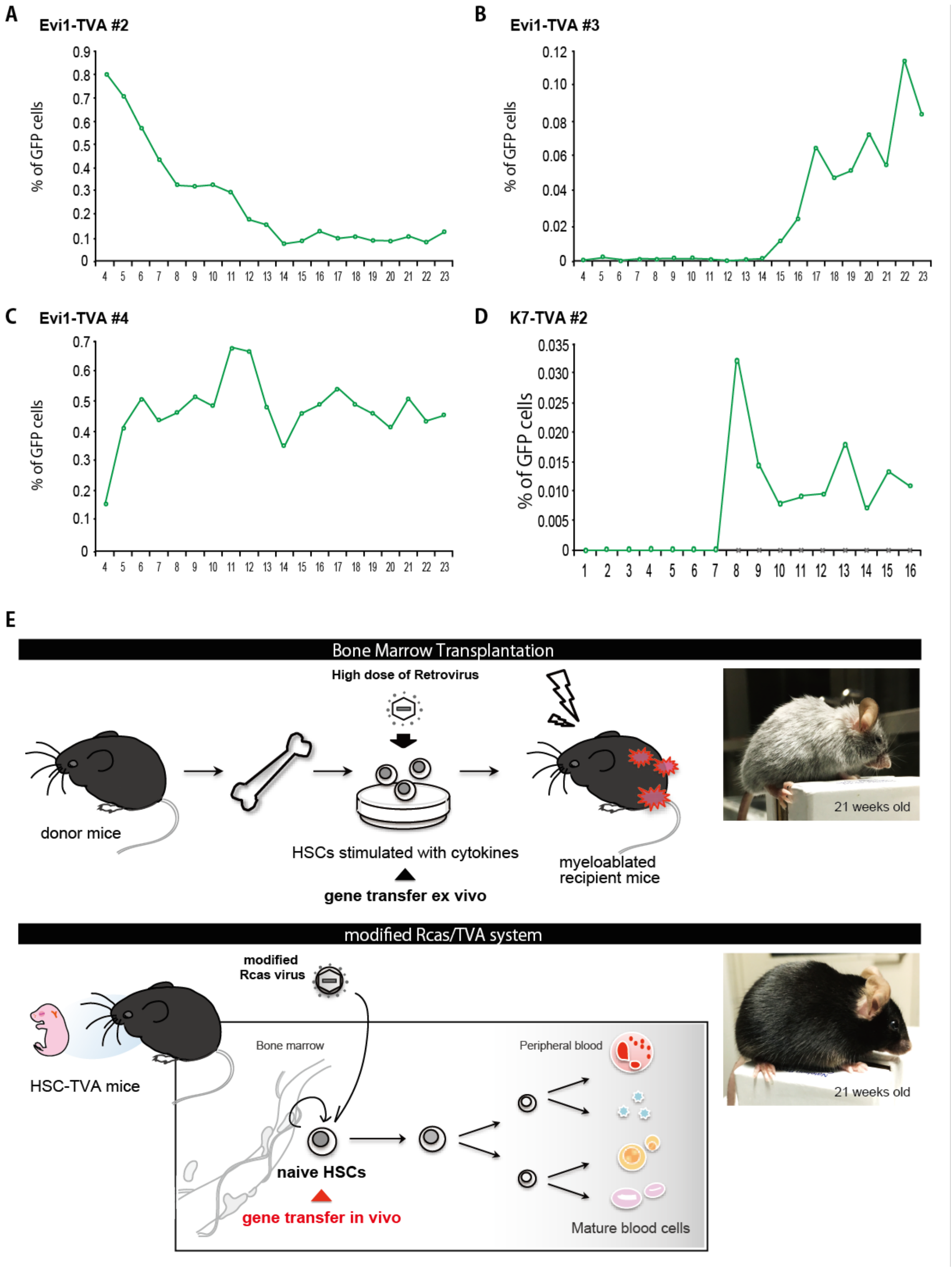

A
Evi1-TVA #2
% of GFP cells
B
Evi1-TVA #3
% of GFP cells
C
Evi1-TVA #4
% of GFP cells
D
K7-TVA #2
% of GFP cells
E
Bone Marrow Transplantation
High dose of Retrovirus
donor mice
HSCs stimulated with cytokines
gene transfer ex vivo
myeloablated recipient mice
21 weeks old
modified Rcas/TVA system
modified Rcas virus
HSC-TVA mice
Bone marrow
Peripheral blood
naive HSCs
gene transfer in vivo
Mature blood cells
21 weeks old

Figure S4, related to Figure 3 and 4: In vivo gene transfer on HSC-TVA mice

(A-C) *In vivo* gene transfer in neonatal Evi1-TVA mice (other individuals to that displayed in Fig.3F). Percentages of GFP^+ cells in the PB of Evi1-TVA mice injected Vs-envA/GFP virus at neonatal stage. **(D)** *In vivo* gene transfer in adult K7-TVA mice (other individual to that displayed in Fig.4C). Percentage of GFP^+ cells in the PB of Krt7-TVA mice injected Vs-envA/GFP virus at adult stage. **(E)** Conceptual diagram of this report. By injecting Rcas retrovirus or modified Vs-envA lentivirus into HSC-TVA mice, transgenes (in our case GFP) carried by the virus are transferred into hematopoietic stem/progenitor cells in vivo. These cells survive long-term and the gene is stably carried on to their progeny (daughter cells), which are found in multiple hematopoietic lineages. Photographs show the appearance of an HSC transplanted mouse following lethal irradiation (above)) and Rcas/GFP virus injected mouse (below). Mice picture were taken by Yoko Tajima.
